# EDUCATION GRADIENTS IN LATER-LIFE COGNITIVE FUNCTION ACROSS LOW-, MIDDLE-, AND HIGH-INCOME COUNTRIES

**DOI:** 10.1093/geroni/igac059.409

**Published:** 2022-12-20

**Authors:** Yuan Zhang, Brendan O'Shea, Xuexin Yu, Tsai-Chin Cho, Kenneth Langa, Alden Gross, Lindsay Kobayashi

**Affiliations:** University of North Carolina at Chapel Hill, Chapel Hill, North Carolina, United States; University of Michigan, Ann Arbor, Michigan, United States; University of Michigan, Ann Arbor, Michigan, United States; University of Michigan School of Public Health, Ann Arbor, Michigan, United States; University of Michigan, Ann Arbor, Michigan, United States; Johns Hopkins Bloomberg School of Public Health, Baltimore, Maryland, United States; University of Michigan, Ann Arbor, Michigan, United States

## Abstract

Education is positively related to cognitive function. However, educational gradients in cognitive function may vary across older populations with different educational compositions and physical and social environments. We conducted one of the first cross-national comparative studies on educational differences in later-life cognitive function using harmonized data. Multivariable linear regressions were employed to estimate the association between education according to International Standard Classification of Education (ISCED) categories and cognitive function for adults ages 60+ from the United States, England, Mexico, South Africa, India, and China. Cross-country differences were tested using fully interacted models. Controlling for demographics and parental education, we found significant educational gradients in cognitive function in low- and middle-income countries; however, in high-income countries, only those with upper secondary education and above had a consistent cognitive advantage over those with primary education. This study suggests substantial country-level differences in cognitive benefits of educational attainment.

